# Supplementary figures and images for: No evidence for enhanced disease with human polyclonal SARS-CoV-2 antibody in the ferret model
Source: PLoS One. 2024 Jun 20;19(6):e0290909. doi: 10.1371/journal.pone.0290909 (PMC11189238; doi:10.1371/journal.pone.0290909)

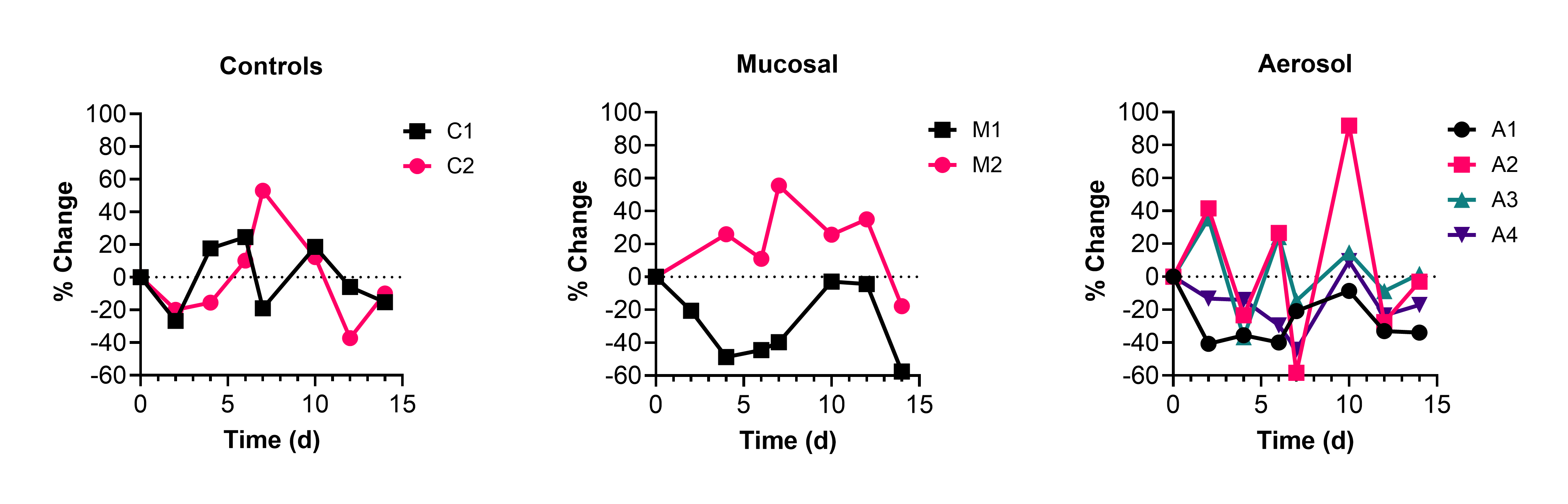

Supplement: S1 Fig — Ferrets were infected by mucosal or aerosol exposure to WA1. At set intervals after infection, respiratory function was recorded using whole-body plethysmography. Left graph shows percent change in respiratory rate for control, uninfected ferrets compared to mucosally-infected ferrets (middle graph) and aerosol-infected ferrets (right graph). Graphs show percent change in respiratory rate with baseline (day 0) for individual ferrets in each group. The numbers in the symbol legends are the individual ferret identification numbers. (TIF) [file pone.0290909.s001.tif]

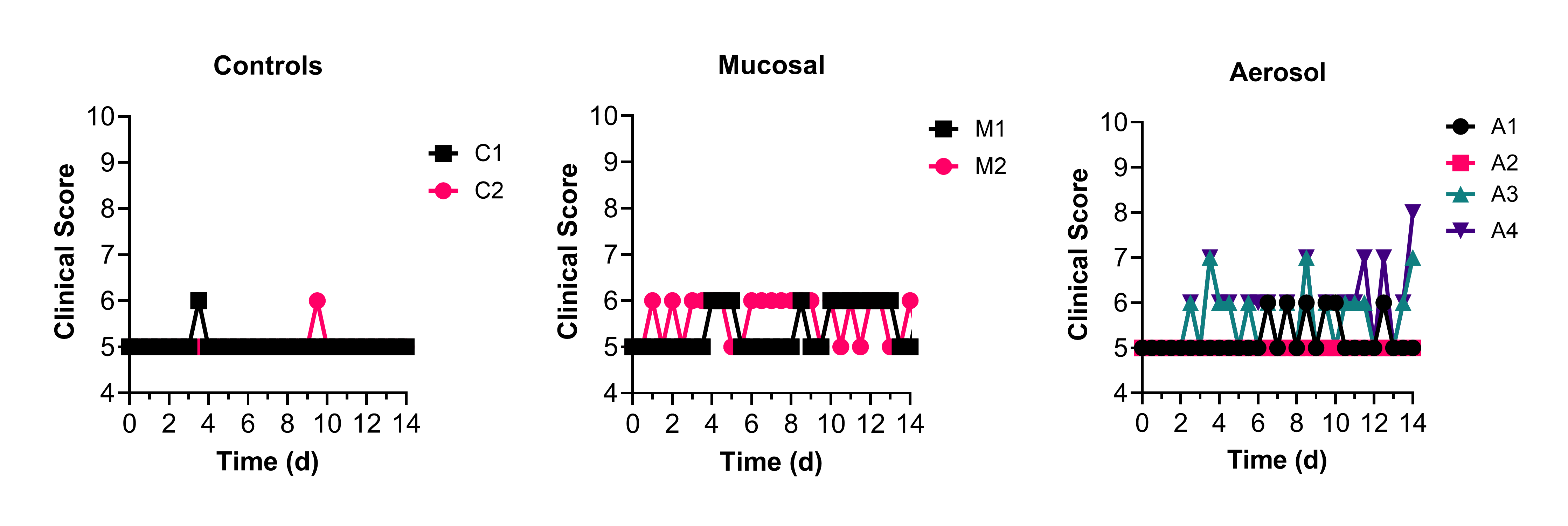

Supplement: S2 Fig — Ferrets were infected by mucosal or aerosol exposure to WA1. At set intervals after infection, with clinical scores recorded twice daily. Left graph shows change in total clinical score for control, uninfected ferrets compared to mucosally-infected ferrets (middle graph) and aerosol-infected ferrets (right graph). The numbers in the symbol legends are the individual ferret identification numbers. (TIF) [file pone.0290909.s002.tif]

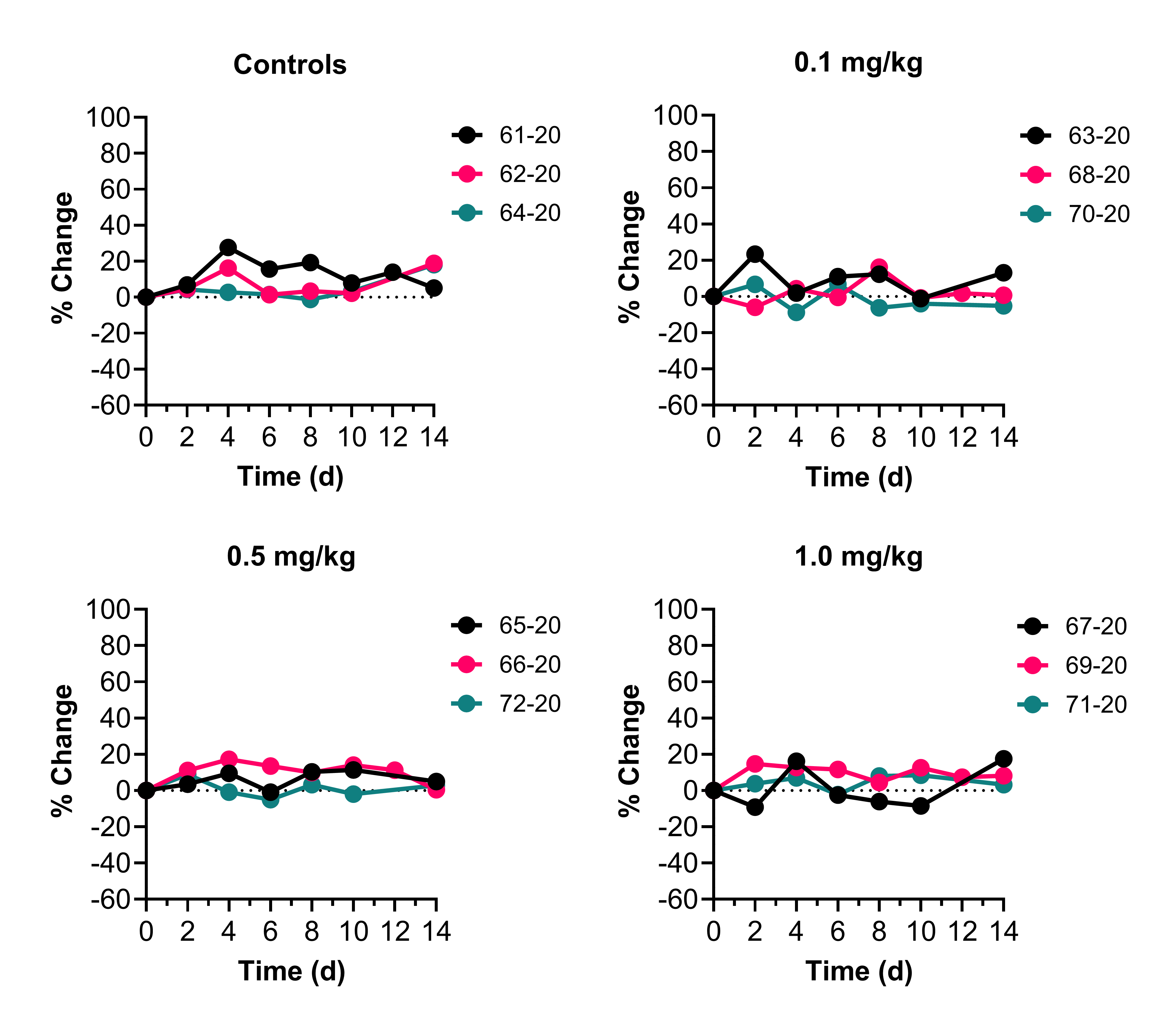

Supplement: S3 Fig — Ferrets were inoculated with SAB185 intravenously 1 day prior to challenge with the Munich virus. Graphs show percent change in respiratory rate with baseline (day 0) for individual ferrets in each group. The numbers in the symbol legends are the individual ferret identification numbers. (TIF) [file pone.0290909.s003.tif]

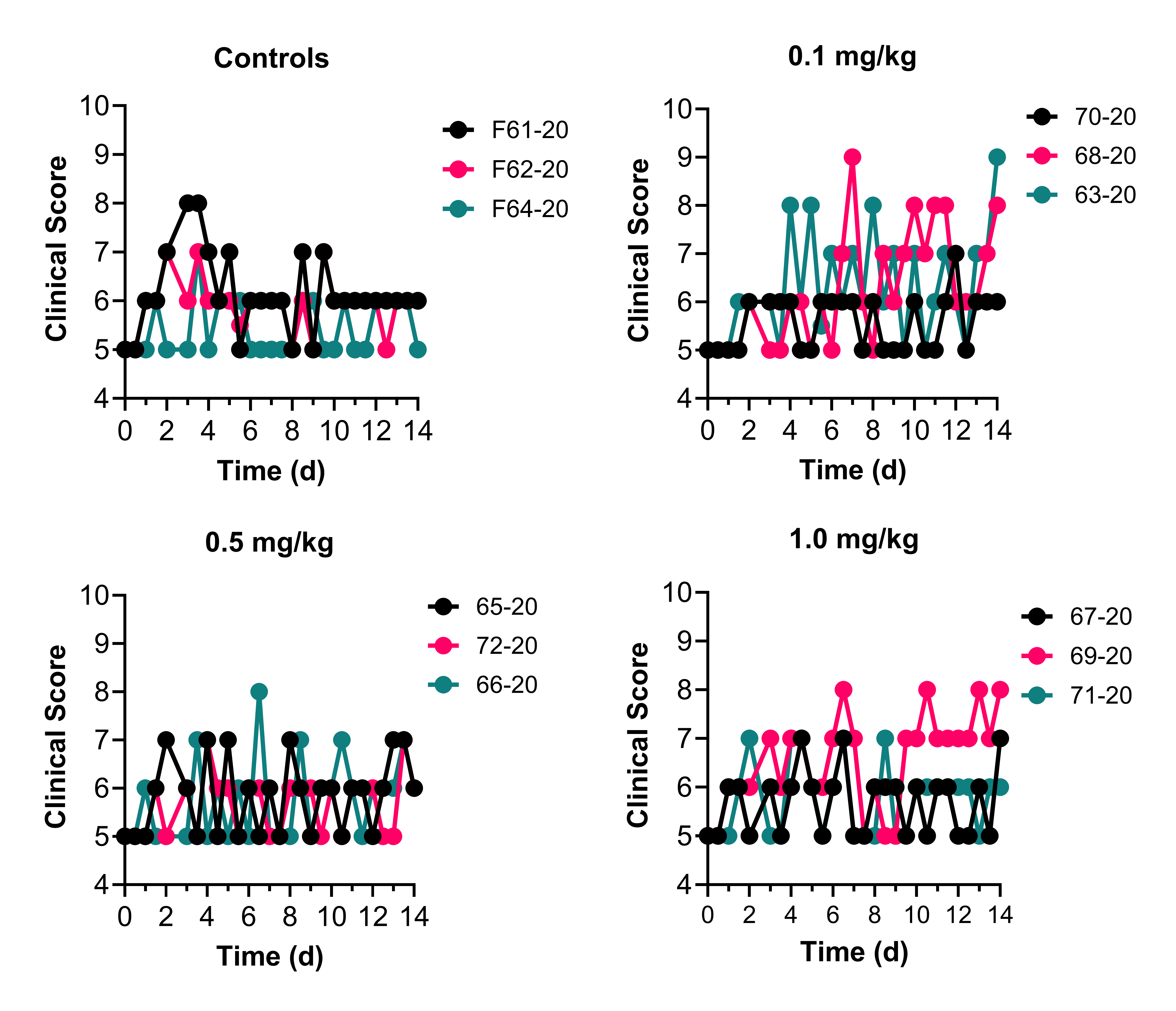

Supplement: S4 Fig — Ferrets were inoculated with SAB185 intravenously 1 day prior to challenge with the Munich virus. Graphs show total clinical scores for individual ferrets in each group. The numbers in the symbol legends are the individual ferret identification numbers. (TIF) [file pone.0290909.s004.tif]
