# Supplementary material for: No evidence for enhanced disease with human polyclonal SARS-CoV-2 antibody in the ferret model
Source: PLoS One. 2024 Jun 20;19(6):e0290909. doi: 10.1371/journal.pone.0290909 (PMC11189238; doi:10.1371/journal.pone.0290909)
Supplement: S1 Table — (DOCX) [file pone.0290909.s005.docx]

**Supplemental Table 1. Clinical Observation Scoring**

Scores for each animal were determined from the matrix below:

|  | **Weight Loss** | **Temperature*** | **Behavior** | **Appearance** | **Respiratory** |
| --- | --- | --- | --- | --- | --- |
| **1** | 1-5% | Normal | Normal | Normal | Normal |
| **2** | 6-10% | Significantly elevated | Less Active | Reduced Grooming | Coughing &/or nasal discharge |
| **3** | 11-15% | Significantly decreased | Huddled | Ruffled Fur | Increased respiration rate &/or labored breathing |
| **4** | 16-20% |  | Only moves when prodded | Hunched |  |
| **Euthanize promptly** | >20% | <33 ^o^C | No response |  | Respiratory distress or rales |

* = significant deviations based on ARIMA modeling of baseline temperature data and time of day

Cumulative scores were determined from addition of a numerical score for each category.  A normal ferret would have a total score a 5 (so a 1 in each category).  Ferrets are observed daily until signs of disease are observed and subsequently, twice daily at least six hours apart. Weights were recorded once daily. Body temperature and activity levels were recorded continuously beginning 3 days prior to infection and continuing until the ferret was euthanized. Body temperature was expected to have normal diurnal variation plus or minus 1-2 deg C from baseline prior to infection.
