# Supplementary material for: No evidence for enhanced disease with human polyclonal SARS-CoV-2 antibody in the ferret model
Source: PLoS One. 2024 Jun 20;19(6):e0290909. doi: 10.1371/journal.pone.0290909 (PMC11189238; doi:10.1371/journal.pone.0290909)
Supplement: S2 Table — Plaque assays were not done on swabs from day 10. Samples are shown as + (positive) or–(negative) rather than a specific titer. Swabs were processed to look for the presence of virus by plaque assay and PCR. Out of concern that virus shedding would be low, near the limit of detection (5 pfu/ml) for plaque assays, plaque assay results from the swabs were scored as positive (+) or negative (-). (DOCX) [file pone.0290909.s006.docx]

**Supplemental Table 2. Plaque assay results for infectious virus in mucosal samples recovered from ferrets after infection with WA1/2020.** Plaque assays were not done on swabs from day 10. Samples are shown as + (positive) or – (negative) rather than a specific titer. Swabs were processed to look for the presence of virus by plaque assay and PCR. Out of concern that virus shedding would be low, near the limit of detection (5 pfu/ml) for plaque assays, plaque assay results from the swabs were scored as positive (+) or negative (-).

| Route | Ferret | ***D2*** | | | ***D4*** | | | ***D7*** | | | ***D14*** | | |
| --- | --- | --- | --- | --- | --- | --- | --- | --- | --- | --- | --- | --- | --- |
|  |  | oral | nasal | rectal | oral | nasal | rectal | oral | nasal | rectal | oral | nasal | rectal |
| Mucosal | M1 | + | + | ─ | + | ─ | ─ | ─ | ─ | ─ | ─ | ─ | ─ |
|  | M2 | ─ | ─ | ─ | + | ─ | ─ | ─ | ─ | ─ | ─ | ─ | ─ |
| Aerosol | A1 | + | + | ─ | ─ | + | ─ | ─ | ─ | ─ | ─ | ─ | ─ |
|  | A2 | + | ─ | ─ | ─ | ─ | ─ | ─ | ─ | ─ | ─ | ─ | ─ |
|  | A3 | ─ | + | ─ | ─ | ─ | ─ | ─ | ─ | ─ | ─ | ─ | ─ |
|  | A4 | + | + | ─ | + | ─ | ─ | ─ | + | ─ | ─ | ─ | ─ |
