# Supplementary material for: No evidence for enhanced disease with human polyclonal SARS-CoV-2 antibody in the ferret model
Source: PLoS One. 2024 Jun 20;19(6):e0290909. doi: 10.1371/journal.pone.0290909 (PMC11189238; doi:10.1371/journal.pone.0290909)
Supplement: S3 Table — (DOCX) [file pone.0290909.s007.docx]

**Supplemental Table 3. Fever data for SAB-185-inoculated and control groups**

|  |  |  | **Fever** | | | **Ave** |
| --- | --- | --- | --- | --- | --- | --- |
| **Group** | **Ferret** | **Sex** | **∆Tmax^a^** | **Duration^b^** | **Fever-Hours^c^** | **Elevation^d^** |
| 1* | F67-20 | M | 1.62 | 84.00 | 69.30 | 0.83 |
|  | F69-20 | F | 1.10 | 48.25 | 30.88 | 0.64 |
|  | F71-20 | F | 1.49 | 57.25 | 44.06 | 0.77 |
|  | **Average** |  | **1.40** | **63.17** | **48.08** | **0.74** |
| 0.5* | F65-20 | F | 1.00 | 34.50 | 22.62 | 0.66 |
|  | F72-20 | F | 1.37 | 40.50 | 27.64 | 0.68 |
|  | F66-20 | M | 1.23 | 40.25 | 28.58 | 0.71 |
|  | **Average** |  | **1.20** | **38.42** | **26.28** | **0.68** |
| 0.1* | F63-20 | F | 1.09 | 6.75 | 4.33 | 0.64 |
|  | F68-20 | F | 1.93 | 44.50 | 41.15 | 0.92 |
|  | F70-20 | M | 1.11 | 20.25 | 13.81 | 0.68 |
|  | **Average** |  | **1.38** | **23.83** | **19.77** | **0.75** |
| Control | F61-20 | M | 1.49 | 21.75 | 16.09 | 0.74 |
|  | F62-20 | M | 1.65 | 30.25 | 26.55 | 0.88 |
|  | F64-20 | M | 1.58 | 28.00 | 23.65 | 0.84 |
|  | **Average** |  | **1.57** | **26.67** | **22.10** | **0.82** |

*dose of SAB-185, in mg/kg

^a^ maximum residual difference in temperature, in degrees Celsius

b fever duration in hours

^c^ sum of significant residual elevations in body temperature, divided by 4 to convert to fever-hours

^d^ average residual difference in temperature, in degrees Celsius
