# Supplementary material for: No evidence for enhanced disease with human polyclonal SARS-CoV-2 antibody in the ferret model
Source: PLoS One. 2024 Jun 20;19(6):e0290909. doi: 10.1371/journal.pone.0290909 (PMC11189238; doi:10.1371/journal.pone.0290909)
Supplement: S4 Table — Samples are shown as + (positive) or–(negative) rather than a specific titer. Swabs were processed to look for the presence of virus by plaque assay and PCR. Out of concern that virus shedding would be low, near the limit of detection (5 pfu/ml) for plaque assays, plaque assay results from the swabs were scored as positive (+) or negative (-). ?+ = questionable positive result. (DOCX) [file pone.0290909.s008.docx]

**Supplemental Table 4. Plaque assay results for infectious virus in mucosal samples from SAB-185 inoculated and control ferrets after challenge with Munich.** Samples are shown as + (positive) or – (negative) rather than a specific titer. Swabs were processed to look for the presence of virus by plaque assay and PCR. Out of concern that virus shedding would be low, near the limit of detection (5 pfu/ml) for plaque assays, plaque assay results from the swabs were scored as positive (+) or negative (-). ?+ = questionable positive result

|  |  | ***D2*** | | | ***D4*** | | | ***D6*** | | | ***D8*** | | | ***D14*** | | |
| --- | --- | --- | --- | --- | --- | --- | --- | --- | --- | --- | --- | --- | --- | --- | --- | --- |
| Group | Ferret | oral | nasal | rectal | oral | nasal | rectal | oral | nasal | rectal | oral | nasal | rectal | oral | nasal | rectal |
| Control | F61 | + | - | - | + | + | - | + | + | - | + | ─ | ─ | ─ | ─ | ─ |
|  | F62 | - | + | - | + | + | - | + | - | - | - | ─ | ─ | ─ | ─ | ─ |
|  | F64 | + | - | - | + | - | - | + | - | - | - | ─ | ─ | ─ | ─ | ─ |
| 0.1 mg/kg | F63 | + | + | - | + | + | - | - | ?+ | - | - | ─ | ─ | ─ | ─ | ─ |
|  | F68 | - | + | - | + | - | - | + | + | - | ?+ | ─ | ─ | ─ | ─ | ─ |
|  | F70 | - | + | - | + | + | - | + | + | - | - | ─ | ─ | ─ | ─ | ─ |
| 0.5 mg/kg | F65 | + | + | - | - | + | - | + | + | - | - | ─ | ─ | ─ | ─ | ─ |
|  | F66 | - | + | - | + | + | - | + | + | - | - | ─ | ─ | ─ | ─ | ─ |
|  | F72 | + | + | - | + | + | - | - | - | - | - | ─ | ─ | ─ | ─ | ─ |
| 1.0 mg/kg | F67 | ?+ | - | - | - | - | - | - | - | - | - | ─ | ─ | ─ | ─ | ─ |
|  | F69 | + | + | - | + | x | - | + | + | ?+ | - | ─ | ─ | ─ | ─ | ─ |
|  | F71 | + | + | - | + | + | - | + | + | ?+ | - | ─ | ─ | ─ | ─ | ─ |
