# Supplementary material for: No evidence for enhanced disease with human polyclonal SARS-CoV-2 antibody in the ferret model
Source: PLoS One. 2024 Jun 20;19(6):e0290909. doi: 10.1371/journal.pone.0290909 (PMC11189238; doi:10.1371/journal.pone.0290909)
Supplement: Attachment — Submitted filename: Reviewer Comments.docx [file pone.0290909.s009.docx]

When submitting your revision, we need you to address these additional requirements.

1. Please ensure that your manuscript meets PLOS ONE's style requirements, including those for file naming. The PLOS ONE style templates can be found at 
<https://journals.plos.org/plosone/s/file?id=wjVg/PLOSOne_formatting_sample_main_body.pdf> and 
<https://journals.plos.org/plosone/s/file?id=ba62/PLOSOne_formatting_sample_title_authors_affiliations.pdf>

2. We noticed you have some minor occurrence of overlapping text with the following previous publication(s), which needs to be addressed:
<https://journals.plos.org/plospathogens/article?id=10.1371%2Fjournal.ppat.1009308>

In your revision ensure you cite all your sources (including your own works), and quote or rephrase any duplicated text outside the methods section. Further consideration is dependent on these concerns being addressed.

Can you be more specific about where the overlapping text is? Other than the ethics statement at the start of the Materials & Methods section – which is boilerplate language - we could not find any overlapping test using the admittedly limited software that we have to compare the two. We do take this seriously and are happy to make changes where warranted, we are just not able to see what is similar between the two outside of the methods section. It should be noted that the paper mentioned includes myself and other members of my laboratory as co-authors. Considering we have not previously published on ferrets, and the other paper is about a different pathogen in cynomolgus macaques using EEG & ICP telemetry (which is not used in this study), it is hard to conceive of any overlap with this other manuscript outside of the methods section.

3. We note that the grant information you provided in the ‘Funding Information’ and ‘Financial Disclosure’ sections do not match.

When you resubmit, please ensure that you provide the correct grant numbers for the awards you received for your study in the ‘Funding Information’ section.

Done. As stated in the Acknowledgements section of the paper, the funding for these studies was provided by the Department of Defense (DOD) and the Biomedical Advanced Research Development Authority (BARDA), not SAB Biotherapeutics. We have made sure our statements in the Funding Information and Financial Disclosure sections reflect this accurately. We did note that in the Declaration of Interests it stated that the work was supported by a DOD contract with SAB. The University of Pittsburgh was contracted by SAB for the animal/virus work, but the money came from the DOD. This has been updated so as to more accurately reflect the funding source.

4. Thank you for stating the following in the Competing Interests section: 
"I have read the journal's policy and the authors of this manuscript have the following competing interests: Thomas Luke, Kristi Egland, Christoph Bausch, Hua Wu, Eddie Sullivan are all employed by SAB Biotherapeutics."

Please confirm that this does not alter your adherence to all PLOS ONE policies on sharing data and materials, by including the following statement: "This does not alter our adherence to PLOS ONE policies on sharing data and materials.” (as detailed online in our guide for authors <http://journals.plos.org/plosone/s/competing-interests>).  If there are restrictions on sharing of data and/or materials, please state these. Please note that we cannot proceed with consideration of your article until this information has been declared.

Done

Please include your updated Competing Interests statement in your cover letter; we will change the online submission form on your behalf.

5. We note that you have stated that you will provide repository information for your data at acceptance. Should your manuscript be accepted for publication, we will hold it until you provide the relevant accession numbers or DOIs necessary to access your data. If you wish to make changes to your Data Availability statement, please describe these changes in your cover letter and we will update your Data Availability statement to reflect the information you provide.

Data will be uploaded to the Open Science Framework repository (OSF), DOI number >>>>>>

6. Please include your full ethics statement in the ‘Methods’ section of your manuscript file. In your statement, please include the full name of the IRB or ethics committee who approved or waived your study, as well as whether or not you obtained informed written or verbal consent. If consent was waived for your study, please include this information in your statement as well.

There were no human subjects in this study. As stated in our ethics section, this work was approved by the University of Pittsburgh’s Institutional Animal Care and Use Committee (IACUC). The polyclonal human antibody product used was made in transgenic cows with human immunoglobulin genes, not obtained from humans.

7. We notice that your supplementary [Supplemental Tables 1-3] are included in the manuscript file. Please remove them and upload them with the file type 'Supporting Information'. Please ensure that each Supporting Information file has a legend listed in the manuscript after the references list.

Done

[Note: HTML markup is below. Please do not edit.]

Reviewers' comments:

Reviewer's Responses to Questions

**Comments to the Author**

1. Is the manuscript technically sound, and do the data support the conclusions?

The manuscript must describe a technically sound piece of scientific research with data that supports the conclusions. Experiments must have been conducted rigorously, with appropriate controls, replication, and sample sizes. The conclusions must be drawn appropriately based on the data presented.

Reviewer #1: Partly

Reviewer #2: Partly

Reviewer #3: Yes

2. Has the statistical analysis been performed appropriately and rigorously?

Reviewer #1: N/A

Reviewer #2: Yes

Reviewer #3: Yes

3. Have the authors made all data underlying the findings in their manuscript fully available?

The [PLOS Data policy](https://nam12.safelinks.protection.outlook.com/?url=http%3A%2F%2Fwww.plosone.org%2Fstatic%2Fpolicies.action%23sharing&data=05%7C01%7Cdsreed%40pitt.edu%7C17fd8c550a8c44f456a208dbcfec9331%7C9ef9f489e0a04eeb87cc3a526112fd0d%7C1%7C0%7C638332387577327123%7CUnknown%7CTWFpbGZsb3d8eyJWIjoiMC4wLjAwMDAiLCJQIjoiV2luMzIiLCJBTiI6Ik1haWwiLCJXVCI6Mn0%3D%7C3000%7C%7C%7C&sdata=P%2FCdei0EB3TTO%2BhYW63BdTtQ1kRSWyalxKi3EPHzD30%3D&reserved=0) requires authors to make all data underlying the findings described in their manuscript fully available without restriction, with rare exception (please refer to the Data Availability Statement in the manuscript PDF file). The data should be provided as part of the manuscript or its supporting information, or deposited to a public repository. For example, in addition to summary statistics, the data points behind means, medians and variance measures should be available. If there are restrictions on publicly sharing data—e.g. participant privacy or use of data from a third party—those must be specified.

Reviewer #1: No

Reviewer #2: Yes

Reviewer #3: Yes

4. Is the manuscript presented in an intelligible fashion and written in standard English?

PLOS ONE does not copyedit accepted manuscripts, so the language in submitted articles must be clear, correct, and unambiguous. Any typographical or grammatical errors should be corrected at revision, so please note any specific errors here.

Reviewer #1: Yes

Reviewer #2: Yes

Reviewer #3: Yes

5. Review Comments to the Author

Please use the space provided to explain your answers to the questions above. You may also include additional comments for the author, including concerns about dual publication, research ethics, or publication ethics. (Please upload your review as an attachment if it exceeds 20,000 characters)

Reviewer #1: The manuscript by Reed et al. submitted to Plos One entitled “No evidence for enhanced disease with polyclonal SARS-CoV-2 antibody in the ferret model.” investigates the potential for antibody-enhanced disease (AED) to SARS-CoV-2 by administering human IgG antibodies into ferrets, followed by virus challenge. The study raises important questions. However, the reviewer feels that the background information/rationale, methods, data presentation, and discussion should be further edited to improve the readability of the manuscript and to support the conclusions drawn.
1. What is known about human IgG (here humanized SAB185 is used) affinity to the ferret FcγR (receptor involved in antibody-dependent cellular cytotoxicity)? While human IgG has great affinity for human FcγR, there are notable species-specific differences in FcγRs. If human IgG has poor affinity for ferret FcγR, then the ferret model may not be a good one to evaluate antibody-enhanced disease in humans. Please provide additional background information for clarity and to support the experimental design and conclusions made here.

We found one paper that examined ferret FcγR binding a human IgG1 mAb, evaluating ADCC enhancement across several species. We also found a paper describing clearance of human influenza mAb within a week of inoculation. However, SAB185 is a polyclonal antibody product so all four IgG subtypes could be represented. We’ve added all of this to the discussion as well as what is known about ADE in dengue fever. We have added to the discussion to say that while in the ferrets SAB185 did not trigger ADE, it remains possible that it would be seen in other models or in humans, or with other antibody treatments for COVID-19.

a. I suggest editing the title to: No evidence for enhanced disease with human polyclonal SARS-CoV-2 antibody in the ferret model.

Done. We agree and thank the reviewer for the suggestion.

2. Phrase “There is also a question of whether SARS-CoV-2 transmission is by contact, droplet, or aerosol” Since aerodynamic size of a respiratory droplet is dynamic, the reviewer suggests avoiding the distinctions of droplet versus aerosol and instead using the term “airborne transmission” (which is inclusive of any size particles traveling through the air) to distinguish it from contact transmission (direct or indirect). PMID: 33977157, 34956601

We have re-written the sentence and replaced droplet/aerosol with airborne, as the reviewer suggested.

3. Methods.
a. What were the ages of the ferrets used in this study? Which sexes were used in which experiments?

Ferrets were young adults (4-7 months in age). In the initial experiment, all ferrets were male. In the second experiment, they were half female, half male. We have added a column to Supplemental Table 2 and added the sex of the ferrets.

b. What was the inoculum dose (pfu/ml)? It was 4x106 pfu for WA1, 8x105 for Munich. We have added this to the mucosal inoculation section of the methods. For WA1 by aerosol, it was 2.3x10^4^ pfu, which was added to the aerosol exposure section.
c. What was the virus diluent used in the nebulizer? What was the humidity and temperature during aerosolization? Virus was in Opti-MEM, although it was not diluted for aerosolization. AGI samples were collected in Opti-MEM with Antifoam A. We have added Opti-MEM in the virology and aerosol exposure sections.
d. Please provide the formula used to calculate the inhaled dose. As stated in the aerosol exposure section of the methods “…inhaled dose was calculated as the product of aerosol concentration of the virus and the accumulated volume of inhaled air, determined as the product of the duration of exposure and the animal’s minute volume.”
e. What was used to anesthetize the animals during telemetry surgery? At what site in the abdomen was the implant placed? How much time did the animals have to recover from surgery before infection? Please clarify the details and timeline. Ferrets were allowed to heal for 2-4 weeks after surgery. We have added this to the methods section.
f. Where is the scoring scale included? It’s not in Supplemental Table 1.

Added and renumbered Supplemental Tables accordingly.

g. What was the limit of detection for genomic RNA and PFU? Please specify in methods or figure legends. The limit of detection for genomic RNA was 1,856 genomes/ml, which we have added to the figure legends as requested. Similarly, the limit of detection for pfu was 5 pfu/ml, which we have added to the legend for Supplemental Tables 2 and 4.

4. Results.
a. Why are the actual infectious virus titers not reported in Table S1? Swabs were processed to look for the presence of virus by plaque assay and PCR. Because we thought that virus shedding would be low, at the limit of detection for plaque assays, we scored the plaque assay results from the swabs as positive (+) or negative (-). We have added this statement to Supplemental Table 2 (Formerly Supplemental Table 1).

b. Was the fever calculated based on a baseline temperature?

Yes, as detailed in the methods section. We have added additional details on how fever was defined.

c. Why was viral RNA tissue distribution done for WA1 but not for Munich SCOV2?
Only a few tissues from a couple of ferrets were positive for virus after infection with Munich.

d. The figures are at very low resolution, and some text is hard to read. Figures provided were TIFF files at 600 dpi. Not sure why they appeared to be low resolution or hard to read. We do agree that in the assembled pdf they are not very nice to look at.

e. Figs. 2 and 6 should have log10 indicated in the Y axis titles.
Done.

f. Fig 3. Is the axis copies/100mg or copies/gram?

We have corrected the legend to reflect what was shown in the figure, which was accurate – copies/100 mg.

g. Please expand and provide more information in the figure legends. The reviewers feels that more information in the legends would allow the reader to understand the graphs without needing to refer to the main text. e.g. Which virus strain and what inoculation route were used in the infection in Figures 4, 5, 6, and 7, this cannot be determined from the legend. What are the numbers associated with symbol legends in figures 4 and 5?

We have made the requested changes, including adding the virus strain and route to Figures 4-7 and indicating that the symbol legends in figures 4 and 5 denote the ferret identification number.

h. Were the WA1/2020 samples collected on days 2, 4, 7, 14 as per table S1? Why is there a day 10 data point in Nasal graph figure 2? And why is not all the data shown? Even if the sample is negative, it could be graphed at or below the limit of detection to show the time course and make it easy to see when the virus was cleared.

Swabs were collected on days 2, 4, 7, 10, and 14. We have added this information to the legends of the figures so it is clear. Samples not plotted had zero plaques or genomes, which cannot be plotted on a log scale graph.

i. What groups do the different colors in Fig. 7 represent?

The different colors represent individual ferrets within the group; we have added that to the legend.

j. What does ?+ mean in table S3? That means it was a questionable positive result. We have added that to the legend for the table (which is now S4).

5. Discussion:
a. “reported here our efforts to develop the ferret as an animal model for COVID-19” The ferret model has been previously used and characterized; other studies report on aerosol inoculations, pathogenesis, transmission data for various SCOV-2 strains, including WA1. The reviewer suggests citing additional relevant manuscripts and discussing the data in the context of similar studies that were conducted in ferrets. For example: PMID: 33827954, 36448801
b. The reviewer appreciates the discussion mentioning influenza virus and studies done in monkeys, but it would be beneficial to contextualize the findings with other studies that conducted aerosol inoculations with both SCOV-2 and flu using the ferret model.

As regards both points, we have re-written the first paragraph in the discussion to focus more on the ferret model of COVID-19 and what others have reported in the published literature.

Reviewer #2: Summary: The manuscript describes a study designed to establish and characterize a ferret model of SARS-CoV-2 infection early in the pandemic, comparing two routes of infection (mucosal and aerosol) and ancestral SARS-CoV-2 that emerged in the human population along with an early variant with a mutation in the spike gene. The authors sought to evaluate whether administration of low doses of a polyclonal human IgG antibody (raised in transchromosomic cattle) against the viral spike protein would cause enhanced disease upon subsequent viral challenge.

Comments: The study seeks to provide important and relevant information (particularly given it was performed early in the pandemic) understanding characteristics of the ferret model and the potential for AED in SARS-CoV-2. The authors address some limitations such as the discrepancy in dose, but for publication the manuscript needs extensive revision to address the following:

• The introduction was nicely written and describes rationale for the study.
• Methods section – would be ideal to provide some more key details even if referred to previously published methods. For example, what was the target for qRT-PCR (so reader doesn’t need to chase down previous manuscripts) and what controls were employed – useful to discuss what negative controls were performed (such as from uninfected ferret tissues) to provide confidence in validity of PCR data and whether ‘genomes/ml or /gram’ is appropriate metric based on what being measured.
additional details on PCR added.

• Need to define what body temperature is defined as a ‘fever’ in ferrets or just describe as spike in temperature (not spike in fever).

Added a description in the methods section of how fever was defined.

• Although the SAB-185 antibody was previously described/characterized, some key details need to be described here as they relate to the study. Here we see no data or description of binding characteristics of this antibody to spike and if neutralizing and the potency that would allow the reader to evaluate whether it is an appropriate candidate for examining AED. It is mentioned offhand once at end of the introduction that this antibody is neutralizing. There is no data shown or described regarding the relationship between levels of antibody administered and virus neutralization in serum (or to show the doses given are sub-neutralizing). There is no description of how the choice of a human IgG might promote AED versus a ferret IgG given some of the known mechanisms of AED.

We have added information on the antibody characteristics that were published in a separate paper which also described work in hamsters (PMID 37736038). Similarly, at the request of reviewer #1, we added information regarding ADE and human IgG in a ferret in the discussion section, along with discussion of other efforts to develop a ferret model for SARS-CoV-2 infection.

• Clinical score and plethysmography were both mentioned in methods but findings were not described nor any data shown.

We have added supplemental figures and described the results in the text.

• Given a focus of the manuscript is enhancement of disease/pathology seen in other examples of respiratory viruses, the pathologic characterization or description of it was inadequate. Need to better discuss what the pathologic changes observed were, if there were inflammatory infiltrates and if these differed in any way in the SAB-185 treated animals.

While we agree with the reviewer that the pathological characterization provided is minimal, the focus of the paper was on enhanced disease. We believe that the other characteristics that were looked at (fever, clinical signs, viral load) as well as the alveolar space analysis provide sufficient information to assess that there is no evidence for enhanced disease at the antibody levels tested.

• Given pathology is not uniform in lungs, need to provide better description of how this was accounted for in regions selected for alveolar space analysis. Similarly, what did the histopathology images shown represent or why were these selected – were these representative of the most severe pathology in each group? Five images of each lung were captured. Regions were chosen at random. The individual who performed the space analysis was blinded to the study and did not know which animals belonged to which group. We added this information to the methods.

Reviewer #3: Reed et al have prepared a well written and clearly presented account of SARS-CoV-2 animal model development, and application to evaluation of an immunotherapy.

The number of animals used is small, which needs to be taken into account in the interpretation of the results, but these data are valuable and informative to the field.

The reprographic quality of the figures is poor, particularly affecting Figure 6 and negating the ability to discern pathology in Figure 7.
We provided 600 dpi tiff images to the journal. We agree that in the assembled pdf the images look poor but we are not certain how to fix that, since the images we provided meet the journal’s criteria and are of good quality.

Minor typographical issues:
Capital M2 on page 19 to replace ‘M1 and m2’ Corrected
Figure 3 – ‘aerosol’ label is split over 2 rows Corrected and revised figure submitted
Page 20: ‘but with a European isolate that had the D614G isolate’. Second ‘isolate’ should be ‘mutation’? Yes, and that is corrected

Minor issues with interpretation of the results:

Page 18: ‘The two mucosally-inoculated ferrets (designated M1 & M2) received a dose of 4x106 pfu; both had a spike in fever within the first 36 hours of infection...’. It is not clear from the figure where M2 has a temperature spike. Consider improving the clarity/visibility of the pink line. Also, provide an explanation for the arrow. You are correct, only M1 had a visible fever in the graph. We have revised the text accordingly.

Page 19: ‘Infectious virus was found in the oral and nasal cavities from all four aerosol-infected ferrets on day 2’. Not all samples were positive, suggest changing to ‘oral and/or nasal’. All four aerosol-infected animals were positive at day 2. For the nasal swab, the data point for A3 and A4 are on top of one another, which makes it harder to see. We have revised Figure 2 to make this more visible.

Page 20: ‘The following morning, all of the ferrets were challenged with 8x105 pfu of Munich-1.1 by mucosal inoculation (IN/oral).’ Please clarify if infection was not as described in the methods which state mucosal inoculation is intranasal, oral and in the eye.

This was corrected to include ocular.

Page 21. ‘Infectious virus was also found in the oral swabs and nasal washes from ferrets in all three groups out to day 8 post-infection (Supplemental Table 3)’. From the table it appears it is all 3 groups out to day 6 and a single positive in the control group at day 8.

Corrected. Also added text to explain that viral RNA was found out to day 8 in nasal and rectal swabs, and oral swabs to day 15 for the control and 0.1 mg/kg groups.

Most significant comment:

Page 24: ‘Our results (fever, weight, viral shedding, pathology), however, would suggest that at least with SAB185 in ferrets at the doses administered, there are no concerns about ADE’.

Regarding 'there are no concerns'. Given the low number of animals used, and the considerable variation in arguably the most important readout (lung pathology/alveolar space) the study is only powered to detect a very large enhancement of disease. The wording in the results ‘no evidence for antibody-enhancement of lung pathology’ is more appropriate. I would propose the discussion applies similar.

Based on comments from reviewer #1, we have modified the discussion and dampened the strength of that statement to say that while we did not see AED in this study it remains possible that it could occur.

6. PLOS authors have the option to publish the peer review history of their article ([what does this mean?](https://nam12.safelinks.protection.outlook.com/?url=https%3A%2F%2Fjournals.plos.org%2Fplosone%2Fs%2Feditorial-and-peer-review-process%23loc-peer-review-history&data=05%7C01%7Cdsreed%40pitt.edu%7C17fd8c550a8c44f456a208dbcfec9331%7C9ef9f489e0a04eeb87cc3a526112fd0d%7C1%7C0%7C638332387577327123%7CUnknown%7CTWFpbGZsb3d8eyJWIjoiMC4wLjAwMDAiLCJQIjoiV2luMzIiLCJBTiI6Ik1haWwiLCJXVCI6Mn0%3D%7C3000%7C%7C%7C&sdata=kXIi21io44xPjsfa9fZc8B85BNMZdkLHUqbk%2FwpDlqc%3D&reserved=0)). If published, this will include your full peer review and any attached files.


If you choose “no”, your identity will remain anonymous but your review may still be made public.


**Do you want your identity to be public for this peer review?** For information about this choice, including consent withdrawal, please see our [Privacy Policy](https://nam12.safelinks.protection.outlook.com/?url=https%3A%2F%2Fwww.plos.org%2Fprivacy-policy&data=05%7C01%7Cdsreed%40pitt.edu%7C17fd8c550a8c44f456a208dbcfec9331%7C9ef9f489e0a04eeb87cc3a526112fd0d%7C1%7C0%7C638332387577327123%7CUnknown%7CTWFpbGZsb3d8eyJWIjoiMC4wLjAwMDAiLCJQIjoiV2luMzIiLCJBTiI6Ik1haWwiLCJXVCI6Mn0%3D%7C3000%7C%7C%7C&sdata=oNasRW7PMYGNuK2THINTuCgBLIRRZRqIbZOzSP1XMzc%3D&reserved=0).

Reviewer #1: No

Reviewer #2: No

Reviewer #3: No
